# Correlation of apparent diffusion coefficient, FAP expression in IHC and [^68^Ga]Ga‑FAPI‑46 uptake using integrated PET/MRI in high-grade soft tissue sarcoma: initial exploratory results

**DOI:** 10.1186/s13550-026-01517-6

**Published:** 2026-09-22

**Authors:** Madelaine Hettler, Franka Menge, Johannes Ludwig, Dominik Nörenberg, Stefan O. Schönberg, Anna Hinterberger, Björn Wängler, Carmen Wängler, Henning Rudolf, Güllü Davarci, Patrick Cieslik, Cristina L. Cotarelo, Melissa Harbrücker, Christoph Reißfelder, Jens Jakob, Wolfgang G. Kunz, Freba Grawe

**Affiliations:** 1 Department of Surgery, Sarcoma Unit, Medical Faculty Mannheim, University Medical Center Mannheim, University of Heidelberg, Mannheim, Germany; 2 Department of Radiology and Nuclear Medicine, Medical Faculty Mannheim, University Medical Center Mannheim, University of Heidelberg, Mannheim, Germany; 3 DKFZ Hector Cancer Institute at the University Medical Center Mannheim, Heidelberg, Germany; 4 Medical Faculty Mannheim, Clinic of Radiology and Nuclear Medicine, Molecular Imaging and Radiochemistry, Heidelberg University, Mannheim, Germany; 5 Medical Faculty Mannheim, Heidelberg University, Research Campus M²OLIE, Mannheim, Germany; 6 Mannheim Institute for Intelligent Systems in Medicine (MIISM), Mannheim, Germany; 7 Medical Faculty Mannheim, Clinic of Radiology and Nuclear Medicine, Biomedical Chemistry, Heidelberg University, Mannheim, Germany; 8 Institute of Pathology, Medical Faculty Mannheim, University Medical Center Mannheim, University of Heidelberg, Mannheim, Germany; 9 Department of Surgery, Medical Faculty Mannheim, University Medical Center Mannheim, University of Heidelberg, Mannheim, Germany; 10 Department of Radiology, University Hospital, Ludwig-Maximilians-University Munich, Munich, Germany; 11 Department of Surgery, Medical Faculty Mannheim, University Medical Center Mannheim, University of Heidelberg, Theodor-Kutzer-Ufer 1-3, 68165 Mannheim, Germany

**Keywords:** Soft Tissue Sarcoma, [^68^Ga]Ga‑FAPI‑46 PET/MRI, Functional Imaging, Treatment response

## Abstract

**Background:**

Fibroblast activation protein inhibitor (FAPI)-targeted positron emission tomography (PET) has emerged as a promising imaging approach in soft tissue sarcomas (STS), whereas magnetic resonance imaging (MRI) remains the diagnostic standard. We evaluated integrated [^68^Ga]Ga‑FAPI‑46 PET/MRI including maximum standard uptake value (SUV_max_), diffusion-weighted imaging, apparent diffusion coefficient (ADC), and contrast-enhanced sequences compared to FAP expression in immunohistochemistry for tumor assessment in high-grade STS.

**Results:**

Thirteen [^68^Ga]Ga‑FAPI‑46 PET/MRI examinations from 9 patients were included, of which 4 were follow-up scans after therapy. All STS showed distinct FAPI uptake (median SUV_max_ 8.4; range 2.9–33). Mean SUV_max_ was 13 (2.9–33) for G2 and 8.4 (7.5–8.4) for G3 tumors. Neither SUV_max_ (*P* = 0.196) nor ADC (*P* = 0.895) differentiated between FNCLCC Grade 2 and Grade 3. A significant inverse correlation was observed between ADC_mean_ and SUV_max_ (Spearman’s ρ = −0.70, 95% CI: −0.93 to − 0.07, *P* = 0.035). Immunohistochemistry demonstrated positive FAP expression in 4 of 9 patients, with significantly higher SUV_max_ in FAP-positive vs. negative tumors in immunohistochemistry (17.7 vs. 6.4; *P* = 0.047). High concordance of [^68^Ga]Ga-FAPI-46 uptake and contrast enhancement was observed. Treatment responders showed marked reductions in SUV_max_ (40–70%) and increased ADC without significant changes in tumor size.

**Conclusion:**

In this exploratory pilot study, [68Ga]Ga-FAPI-46 PET/MRI provided complementary functional information in high-grade STS. Although no correlation with grading was observed either using SUVmax or ADC, a significant inverse correlation between ADCmean and SUVmax was identified within this cohort. Preliminary observations regarding treatment monitoring suggested that early changes in tracer uptake and ADC may reflect treatment-related changes, however, these findings were derived from a small pilot cohort. Larger prospective studies are required to validate these observations and to further define the potential role of [68Ga]Ga-FAPI-46 PET/MRI in treatment monitoring and response assessment.

## Introduction

Soft tissue sarcomas (STS) represent a rare and heterogeneous group of malignant mesenchymal neoplasms. Over 80 different histological subtypes have been identified, with liposarcomas (LPS) and leiomyosarcomas (LMS) being the most prevalent entities [1]. Precise tumor characterization is essential for both prognosis and treatment planning; in particular, tumor grading plays a key role [2]. While primary surgical resection remains the standard of care for localized, non-metastatic low-grade STS, neoadjuvant treatment strategies such as radiotherapy and/or chemotherapy are considered for high-grade STS to improve tumor control [3]. For primary tumor assessment, magnetic resonance imaging (MRI) is preferred over computed tomography (CT) due to its superior soft tissue contrast and lack of ionizing radiation [4]. Diagnosis is subsequently confirmed by histopathological evaluation of a tumor specimen. Tumor grading is based on the classification system of the French Fédération Nationale des Centres de Lutte Contre le Cancer (FNCLCC). However, the reliability of histopathological grading may be limited in large and heterogeneous STS, particularly when core needle biopsies are used, as these may not adequately capture the full histological spectrum of the tumor [5, 6]. To address these limitations, imaging parameters, primarily from MRI, are increasingly integrated into the evaluation and grading of STS to support therapeutic decision-making. In particular, peritumoral contrast enhancement, presence of intratumoral necrosis, and marked signal heterogeneity on dynamic contrast-enhanced MRI have been proposed to differentiate between low-grade and high-grade STS [7]. In addition, the apparent diffusion coefficient (ADC) derived from diffusion-weighted imaging (DWI) has shown correlation with FNCLCC grading and may serve as a quantitative imaging biomarker [8–10].

^18^F-fluorodeoxyglucose positron emission tomography ([^18^F]FDG PET/CT) has gained recognition as a functional imaging modality for staging, recurrence, treatment response assessment, and prognostication in sarcomas [11]. However, in STS, its role remains limited. Although elevated standardized uptake values (SUV) from [^18^F]FDG PET have been associated with high-grade disease, [^18^F]FDG uptake lacks specificity due to overlapping metabolic activity across different histological subtypes and grades [12, 13]. Consequently, [^18^F]FDG PET is not routinely recommended for STS grading or treatment monitoring. Fibroblast activation protein (FAP) has recently emerged as a promising target for molecular imaging. FAP is a cell surface serine protease highly expressed by cancer-associated fibroblasts (CAFs), which are abundant in the tumor stroma of many epithelial malignancies, but largely absent in normal tissue [14, 15].

Novel FAP-targeted PET tracers have demonstrated high tumor-to-background contrast across a broad spectrum of cancers, including sarcomas [16–18]. In contrast to most epithelial malignancies, sarcomas frequently exhibit FAP expression not only in CAFs but also directly in tumor cells, consistent with their mesenchymal origin. This distinct expression pattern has been reported across multiple soft-tissue and bone sarcoma subtypes and provides a biological rationale for FAP-targeted imaging in STS [19]. In this exploratory cohort, we systematically evaluated integrated [^68^Ga]Ga-FAPI-46 PET/MRI in G2 and G3 STS by correlating PET-derived [^68^Ga]Ga-FAPI-46 uptake with multiparametric MRI and histopathological FAP expression for STS assessment. Previous studies have investigated the relationship between FAPI PET uptake and histopathological FAP expression [16, 20, 21]. To our knowledge, however, the present study is the first to directly integrate [^68^Ga]Ga-FAPI-46 PET with multiparametric MRI—the radiological reference standard for soft-tissue sarcoma—and histopathological tumor characteristics within the same cohort.

## Materials and methods

### Study design and patient population

A total of 26 patients underwent [^68^Ga]Ga-FAPI-46 PET/MRI for staging and/or treatment response assessment between May 2024 and May 2025. For this study, nine patients with STS were included, comprising five patients with a primary tumor and four with a local recurrence. All of these baseline scans were used to assess tumor characterization parameters. Where applicable, an additional follow-up [^68^Ga]Ga-FAPI-46 PET/MRI scan was performed after completion of neoadjuvant therapy (*n* = 4) and included for treatment response evaluation. In total, 13 [^68^Ga]Ga-FAPI-46 PET/MRI examinations were included in the final analysis.

Patients were referred for additional imaging based on clinical indication by their treating physician. All patients gave written consent to undergo [^68^Ga]Ga-FAPI-46 PET/MRI according to the regulations of the German Pharmaceuticals Act § 13(2b) under the direct responsibility of the applying physician. This retrospective analysis of the data, which was part of an ongoing prospective registry study, was approved by the local ethics committee (EK II 2023-886-AF 11). Written informed consent for participation was obtained from all participants. The study was conducted in compliance with the ethical principles of the Declaration of Helsinki and its later amendments.

### Radiosynthesis

The radiosynthesis was performed using a GRP synthesis module (Scintomics, Fürstenfeldbruck, Germany). In brief, 50 µg of FAPI-46 precursor (SOFIE Biosciences, Virginia, USA) and 3 mg of ascorbic acid were dissolved in 500 µL of water for injection to which were added 100 µL of sodium acetate buffer (3 M, pH 4.8). After transferring the FAPI-46 solution to a reaction vial, [^68^Ga]GaCl_3_, which was obtained from a commercially available ^68^Ge/^68^Ga generator system (GalliAd, 1.85 GBq, IRE-ELiT, Belgium), was eluted directly into the reaction vial, resulting in a pH of 4.0. The reaction mixture was heated to 140 °C for 7 min. The product was purified using a Sep-Pak C18 Light cartridge, eluted with 1 mL of EtOH/water (1:1), and diluted with 7 mL of PBS through a sterile filter into a product vial, giving the radioligand in injectable form.

### Image acquisition

No specific patient preparation was required. A mean activity of 186 ± 46 MBq [^68^Ga]Ga-FAPI-46 was administered intravenously. Image acquisition started approx. 60 min post injection. Whole-body PET/MRI (skull base to mid-thigh) was performed on a hybrid Biograph mMR 3.0-T scanner (Siemens Healthineers) in head-first prone position. PET data were reconstructed using an HD PET algorithm with 3 iterations and 21 subsets, a matrix size of 172 × 172, and a slice thickness of 2 mm. Attenuation correction was based on the vendor-provided MRAC CAIPI HiRes sequence. The multiparametric (mp) MRI protocol consisted of T2-HASTE, DWI with ADC mapping using an axial echo-planar sequence (b values: 50, 800 s/mm², section thickness 5 mm), and contrast-enhanced T1-weighted imaging (DCE-MRI).

### Image analysis

PET analysis comprised both visual and semiquantitative evaluation. Mean and maximum standard uptake values (SUV_mean/max_) normalized to body weight were with dedicated software (syngo.via, Siemens Healthineers). For patient-level analyses, the highest lesion SUV_max_ per patient was used. Tracer uptake was considered positive if focal activity was visually discernible above adjacent background. From the axial ADC map, the visually darkest intratumoral area (lowest diffusion) was manually identified as ROI, and ADC_mean_, ADC_min_, and ADC_max_ were determined with an ellipsoidal measurement tool (1 cm² area). ADC analysis was performed using ADC_mean_ values, as this parameter is consistently reported in the literature and enables comparability with existing analyses [9]. Tumor size and contrast enhancement were assessed on T1 post contrast MRI sequences. All images were independently reviewed by one experienced nuclear medicine physician/radiologist and one sarcoma surgeon. Discrepancies were resolved by consensus. Treatment response was categorized as complete/partial metabolic response (CMR/PMR), stable metabolic disease (SMD) and progressive metabolic disease (PMD) using SUV_max_-based mPERCIST criteria based on baseline and follow-up imaging at 4–12 weeks. Metabolic response was descriptively compared with morphologic response according to RECIST 1.1.

### Histopathological findings

Tumor tissue was obtained either from diagnostic core needle biopsies (in patients receiving neoadjuvant therapy) or from surgical resection specimens (in patients undergoing upfront surgery) and was processed according to institutional standards for FAP analysis. Owing to the retrospective design of the study, no additional image-guided biopsies were obtained following [⁶⁸Ga]Ga-FAPI-46 PET/MRI. Consequently, all FAP IHC assessments were performed on the original tumor specimens available at initial diagnosis. For recurrent cases, analysis was conducted on tissue from the recurrence.

The specimens were stained with standard hematoxylin and eosin and FAP immunohistochemistry (IHC). As primary anti-FAP antibody, ab207178 (EBR20021; Abcam, Cambridge, UK) was used. Slides were evaluated under light microscopy with 40x power by one experienced pathologist (CC). Histopathological grading was performed according to the FNCLCC criteria. FAP expression was reviewed in accordance with a previously described scoring system [22].

As tissue sampling was performed independently of the imaging examinations and was not specifically targeted to PET- or MRI-defined regions, precise spatial matching between the histopathological findings and imaging-derived parameters could not be achieved. Consequently, the pathological assessment represents a patient-level tissue sample considered representative of the tumor but not necessarily the region corresponding to the highest tracer uptake on [^68^Ga]Ga-FAPI-46 PET/MRI.

### Statistical analysis

All quantitative analyses for tumor characterization were performed at the patient level using the baseline [^68^Ga]Ga-FAPI-46 PET/MRI examination of each patient (*n* = 9). Follow-up examinations acquired after neoadjuvant therapy (*n* = 4) were not included in these analyses and were evaluated exclusively for treatment response assessment using SUV_max_-based modified PERCIST. Statistical analyses were conducted using jamovi software (version 2.6.26.0). Descriptive statistics are reported as frequencies and percentages for categorical variables and as medians with ranges for continuous variables. Data distribution was assessed using the Shapiro–Wilk test. Correlations between imaging-derived and histopathological parameters (grading, ADCmean, SUVmax, and FAP expression) were evaluated using Pearson’s correlation coefficient for normally distributed continuous variables and Spearman’s rank correlation coefficient for ordinal or non-normally distributed variables. Correlation coefficients are reported together with their corresponding 95% confidence intervals (CI). Statistical significance was defined as a two-sided P-value < 0.05. Given the exploratory nature of this pilot study and the limited sample size, no adjustment for multiple comparisons was performed. Therefore, all statistical analyses should be considered hypothesis-generating. Tables and boxplots for data visualization were generated using Microsoft Excel for Mac (version 16.80; Microsoft Corp., Redmond, WA, USA).

## Results

### Patient and Tumor Characteristics

Thirteen [^68^Ga]Ga-FAPI-46 PET/MRI scans obtained from nine patients (two female; median age 64 years, range, 55–79) with histologically confirmed non-metastatic STS were analyzed (Table 1). Of these, nine scans were performed for initial staging of the primary tumor (*n* = 5) or local recurrence (*n* = 4), and four scans for additional early response assessment at three months follow-up after (neoadjuvant) treatment, including radiotherapy (*n* = 2), chemotherapy (*n* = 1), and FAPI-directed radioligand therapy (*n* = 1) (Table 1). Median lesion size was 9.4 cm (2.5–37.0 cm). Histological subtypes comprised 6 dedifferentiated liposarcomas (DDLPS), one undifferentiated pleomorphic sarcoma (UPS), one myxofibrosarcoma (MFS), and one fat-forming solitary fibrous tumor (SFT). Tumor grading (G) according to the FNCLCC system classified five patients with G2 and three patients with G3 STS. SFT was classified as GX, as FNCLCC grading is not applicable to this histological subtype.

**Table 1:** Patient, Tumor, and Imaging Characteristics

Patient No.	Age (years)	Sex	Tumor location	Tumor Manifestation	Histological Subtype	Tumor Size (cm)	Grading (FNCLCC)	Multi-focality	Treatment history	Contrast enhancement on MRI	ADC_mean_ (mean ± SD) [×10-³ mm²/s]	[^68^Ga]Ga‑FAPI‑46 uptake (SUV_max_)	FAP expression in IHC	FAP IHC Score
**01**	68	male	Retroperitoneum	PT	DDLPS	37.0	G3	No	Surgery	positive	0.698 ± 0.333	pos (8.4)	negative	0
**02**	73	male	Retroperitoneum	PT	DDLPS	9.6	G2	No	Surgery	positive	0.741 ± 0.033	pos (33.0)	positive	+ 3
**03**	57	male	Retroperitoneum	LR	DDLPS	8.8	G2	Yes	[¹⁷⁷Lu]Lu-FAPI-46 Therapy	positive	0.783 ± 0.063	pos (8.8)	negative	0
**04**	58	male	Retroperitoneum	PT	UPS	9.4	G2	No	RT + CHT + Surgery	positive	1.001 ± 0.067	pos (13.0)	positive	+ 3
**05**	55	female	Lower extremity	PT	MFS	4.2	G2	No	RT + Surgery	positive	0.814 ± 0.165	pos (16.0)	positive	+ 3
**06**	59	male	Lower extremity	LR	DDLPS	4.3	G2	No	CHT + Surgery	positive	1.268 ± 0.055	pos (2.9)	negative	0
**07**	68	male	Retroperitoneum	LR	Fat Forming SFT	11.5	Gx	No	RT + Surgery	positive	1.926 ± 0.174	pos (4.3)	negative	0
**08**	79	female	Retroperitoneum	PT	DDLPS	13.5	G3	No	RT + Surgery	positive	1.065 ± 0.088	pos (7.5)	negative	0
**09**	64	male	Retroperitoneum	LR	DDLPS	2.5	G3	Yes	RT + Surgery	positive	1.074 ± 0.216	pos (8.4)	positive	+ 3

ADC: Apparent diffusion coefficient; FAPI: Fibroblast Activation Protein Inhibitor, FAP: Fibroblast Activation Protein, IHC: Immunohistochemistry; PT: Primary tumor; LR: Local recurrence; DDLPS: Dedifferentiated liposarcoma; UPS: Undifferentiated pleomorphic sarcoma, MFS: Myxofibrosarcoma; SFT: Solitary fibrous tumor, G: Grade; RT: Radiotherapy; CHT: Chemotherapy

### STS lesion assessment

**Fig. 1:**
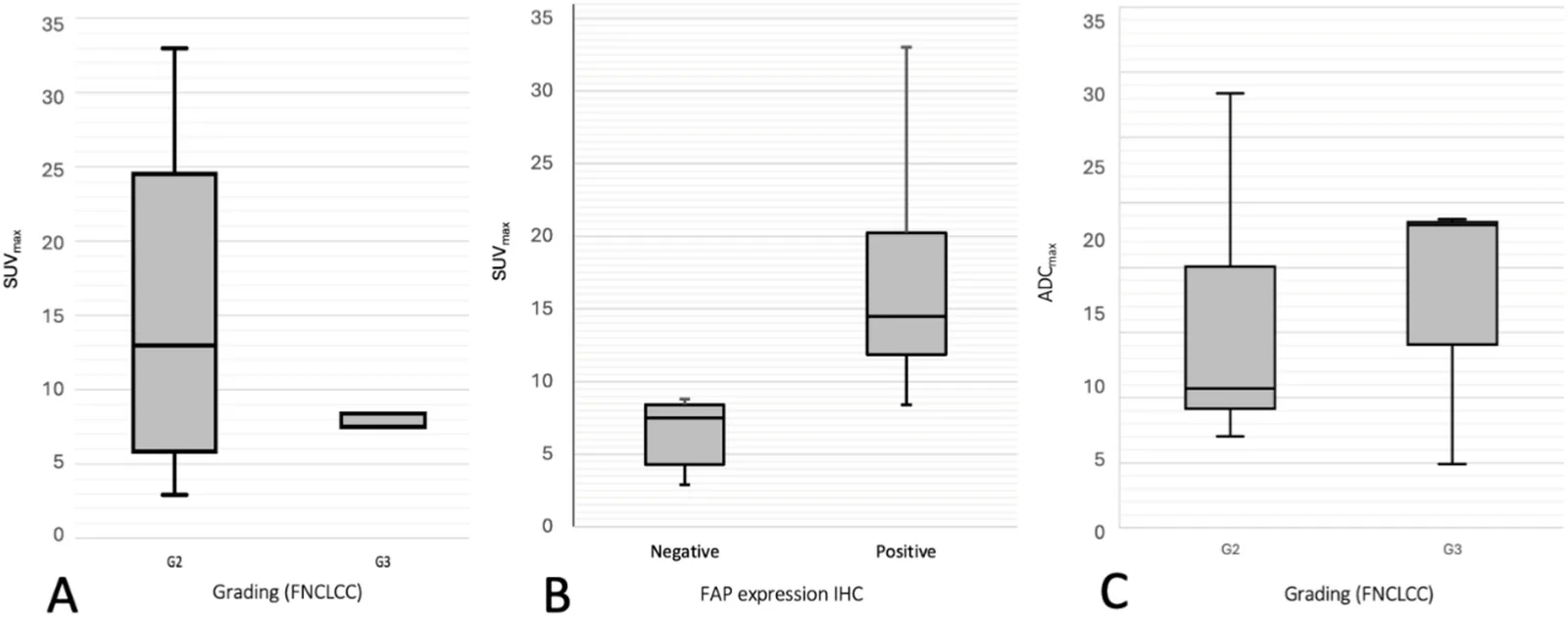
Boxplots showing the distribution of [^68^Ga]Ga-FAPI-46 uptake, immunohistochemical FAP status, FNCLCC Grading and ADC_mean_. **A**: Non-significant correlation of [68Ga]Ga-FAPI-46 uptake in PET/MRI with FNCLCC Grading (Spearman’s ρ = 0.51, 95% CI: −0.25 to 0.87, *P* = 0.196). **B**: Comparison of immunohistochemical FAP status with corresponding SUV_max_ from [^68^Ga]Ga-FAPI-46 PET/MRI shows significantly lower tracer uptake in FAP-negative tumors (SUV_max_ 6.38) compared to FAP-positive tumors (SUV_max_ 17.7; Spearman’s ρ = 0.713, 95% CI: 0.09–0.93, *P* = 0.047). **C**: Distribution of ADC values with FNCLCC grading shows ADC_mean_ for FNCLCC G2 tumors of 0.921 ± 0.077 × 10-³ mm²/s and 0.945 ± 0.176 × 10-³ mm²/s for G3 tumors without significance (Spearman’s ρ =-0.056, 95% CI: −0.71 to 0.67, *P* = 0.895)

All STS lesions demonstrated clear [^68^Ga]Ga-FAPI-46 uptake with a median SUV_max_ of 8.4 (range, 2.9–33; see Table 1) and a positive contrast enhancement on MRI across all histological subtypes. The median SUV_max_ was 13 (2.9–33) for FNCLCC G2 and 8.4 (7.5–8.4) for G3 with no statistically significant correlation between SUV_max_ and FNCLCC grading (Spearman’s ρ = 0.51, 95% CI: −0.25 to 0.87, *P* = 0.196). [^68^Ga]Ga-FAPI-46 uptake in G3 tumors was consistent within a moderate range (7.5–8.4), whereas G2 STS demonstrated a broader distribution of uptake values (Fig. 1A). This included cases with very high and homogeneous uptake (SUV_max_ 33.0), as well as lesions with minimal tracer uptake (SUV_max_ 2.9). Both the highest and the lowest SUV_max_ were observed in DDLPS, reflecting considerable heterogeneity within this subgroup. No difference in tracer uptake among histological subtypes was found.

**Fig. 2:**
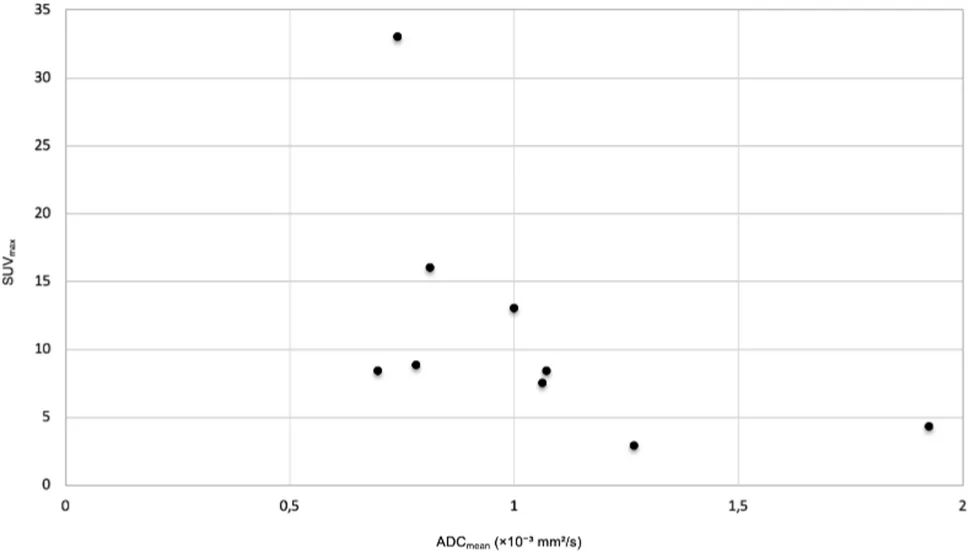
Scatterplot illustrating the relationship between ADC_mean_ and SUV_max_ from [^68^Ga]Ga-FAPI-46 PET/MRI. A significant inverse correlation was observed (Spearman’s ρ = -0.70, 95% CI: −0.93 to − 0.07, *P* = 0.035)

ADC_mean_ for G2 was 0.921 ± 0.077 × 10^−^³ mm²/s and 0.945 ± 0.176 × 10^−^³ mm²/s for G3 without significant association between ADC values and tumor grade (Spearman’s ρ = −0.056, 95% CI: −0.71 to 0.67, *P* = 0.895). Using the dichotomous model, which summarizes both G2 and G3 as high-grade STS, the mean ADC is 0.930 ± 0.114 × 10^−^³ mm²/s. These findings indicate a substantial overlap of ADC values between G2 and G3, with very similar median values across both groups (Fig. 1C). A significant inverse correlation was observed between ADC_mean_ and SUV_max_ (Spearman’s ρ = −0.70, 95% CI: −0.93 to − 0.07, *P* = 0.035; Fig. 2).

FAP expression was assessed by IHC. Positive FAP expression was detected in 4/9 cases. The remaining 5 tumors showed no detectable expression (IHC score 0), although tracer uptake was observed on PET imaging. FAP-negative patients included retroperitoneal DDLPS (*n* = 1 G2, *n* = 2 G3), retroperitoneal SFT (Gx), and DDLPS (G2) of the lower extremity. All tumors with positive FAP immunoreactivity demonstrated a strong expression with IHC score of + 3 (Table 1). Comparison of FAP status in IHC with corresponding SUV_max_ values from PET revealed a significant lower tracer uptake in FAP-negative tumors (SUV_max_ 6.4) and higher uptake (SUV_max_ 17.7) in FAP-positive tumors (Spearman’s ρ = 0.713, 95% CI: 0.09 to 0.93, *P* = 0.047; Fig. 1B).

**Fig. 3:**
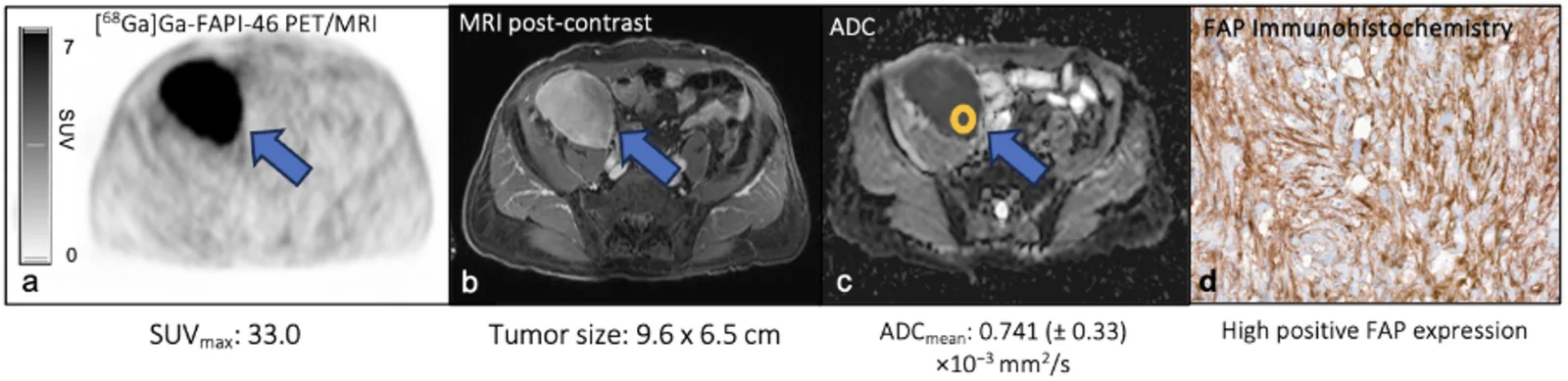
Patient with primary retroperitoneal dedifferentiated liposarcoma and matched marked findings in [^68^Ga]Ga‑FAPI‑46 PET, MRI and IHC. (**a**) PET imaging shows a homogeneous and intense [^68^Ga]Ga‑FAPI‑46 uptake of the sarcoma (SUV_max_ 33.0). (**b**) Corresponding marked and homogeneous contrast enhancement on MRI, with restricted diffusion (not shown). (**c**) ADC map reveals low *ADC*_*mean*_ value (0.741 ± 0.33 × 10⁻³mm²/s); yellow circle indicates the ROI used for ADC measurement. (**d**) FAP immunohistochemistry shows high positive FAP expression

**Fig. 4:**
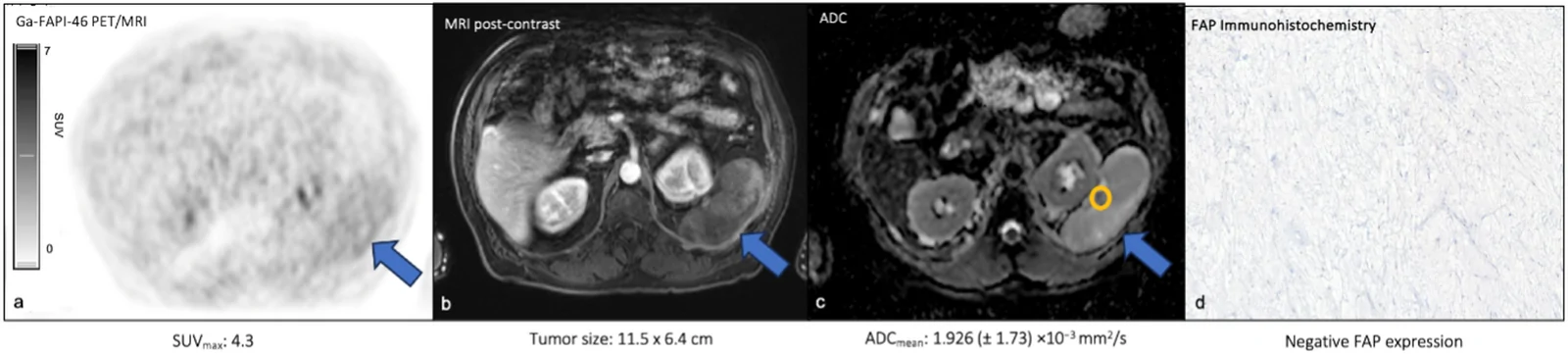
Patient with retroperitoneal fat-forming SFT and matched low FAPI- uptake, low contrast-enhancement, and negative FAP expression in IHC. (**a**) PET shows very low [^68^Ga]Ga‑FAPI‑46 uptake of the tumor (blue arrow; SUV_max_ 4.3) with (**b**) correlating minimal contrast enhancement on MRI. (**c**) Diffusion restriction is low (not shown) with a high *ADC*_*mean*_ (1.926 ± 1.73 × 10⁻³mm²/s); yellow circle indicates the ROI used for ADC measurement. (**d**) Immunohistochemistry also shows negative FAP expression in IHC

**Fig. 5:**
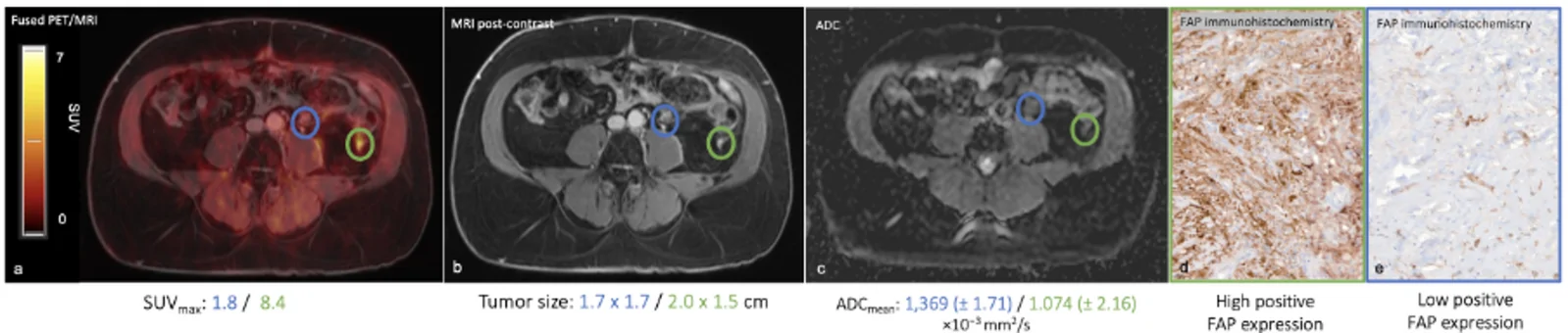
Patient with multifocal recurrent retroperitoneal dedifferentiated liposarcoma (G3) and matched imaging findings. (**a**) Multifocal soft tissue sarcoma presents with one lesion (green circle) showing high [^68^Ga]Ga‑FAPI‑46 uptake (SUV_max_ 8.4) and high positive FAP expression in immunohistochemistry (**d**) as well as strong contrast enhancement in MRI (**b**). Another lesion (blue circle) shows low FAPI uptake (SUV_max_ 1.8) in PET (**a**) and low FAP expression in immunohistochemistry (**e**) but moderate contrast enhancement in MRI (**b**). The lesion with high FAPI uptake (green circle) also exhibits a lower *ADC*_*mean*_ value compared to the low-uptake lesion (blue circle) (**c**)

DDLPS, the most prevalent subtype, exhibited a median SUV_max_ of 8.4 (2.9–33) with substantial variability in uptake patterns. Examples ranged from uniformly intense FAPI uptake in a primary retroperitoneal DDLPS G2 (SUV_max_ 33; Fig. 3) to consistently low uptake in a fat-forming SFT with intermediate malignancy (SUV_max_ 4.3; Fig. 4), each mirroring the corresponding MRI contrast enhancement. Pronounced intratumoral and intrapatient heterogeneity was evident in a multifocal recurrent retroperitoneal DDLPS G3, which presented with one lesion showing high uptake (SUV_max_ 8.4) and another with minimal uptake on both PET (SUV_max_ 1.8) and contrast-enhanced MRI (Fig. 5).

### Evaluation of treatment response

In total, four patients underwent [^68^Ga]Ga-FAPI-46 PET/MRI for response assessment.

**Fig. 6:**
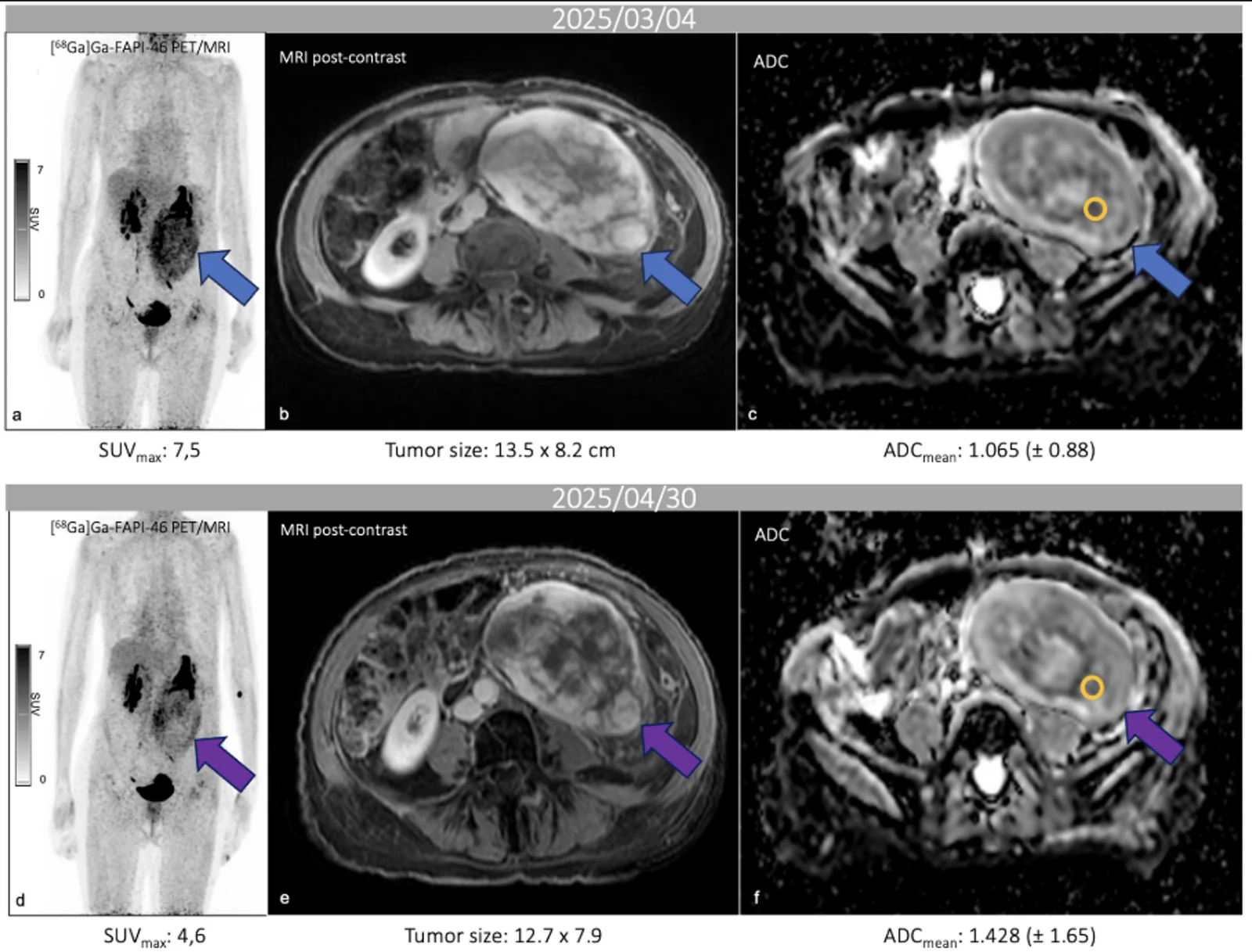
[^68^Ga]Ga‑FAPI‑46 PET/MRI before (**a-c**) and after (**d-f**) neoadjuvant radiotherapy of a high-grade retroperitoneal DDLPS. Imaging demonstrates a marked decrease in SUV_max_, accompanied by an increase in ADC_mean_ with only slight decrease in tumor size, overall indicating treatment response. Yellow circle indicates the ROI used for ADC measurement. Histopathological correlation was not possible, as the patient declined surgical treatment following neoadjuvant therapy

**Fig. 7:**
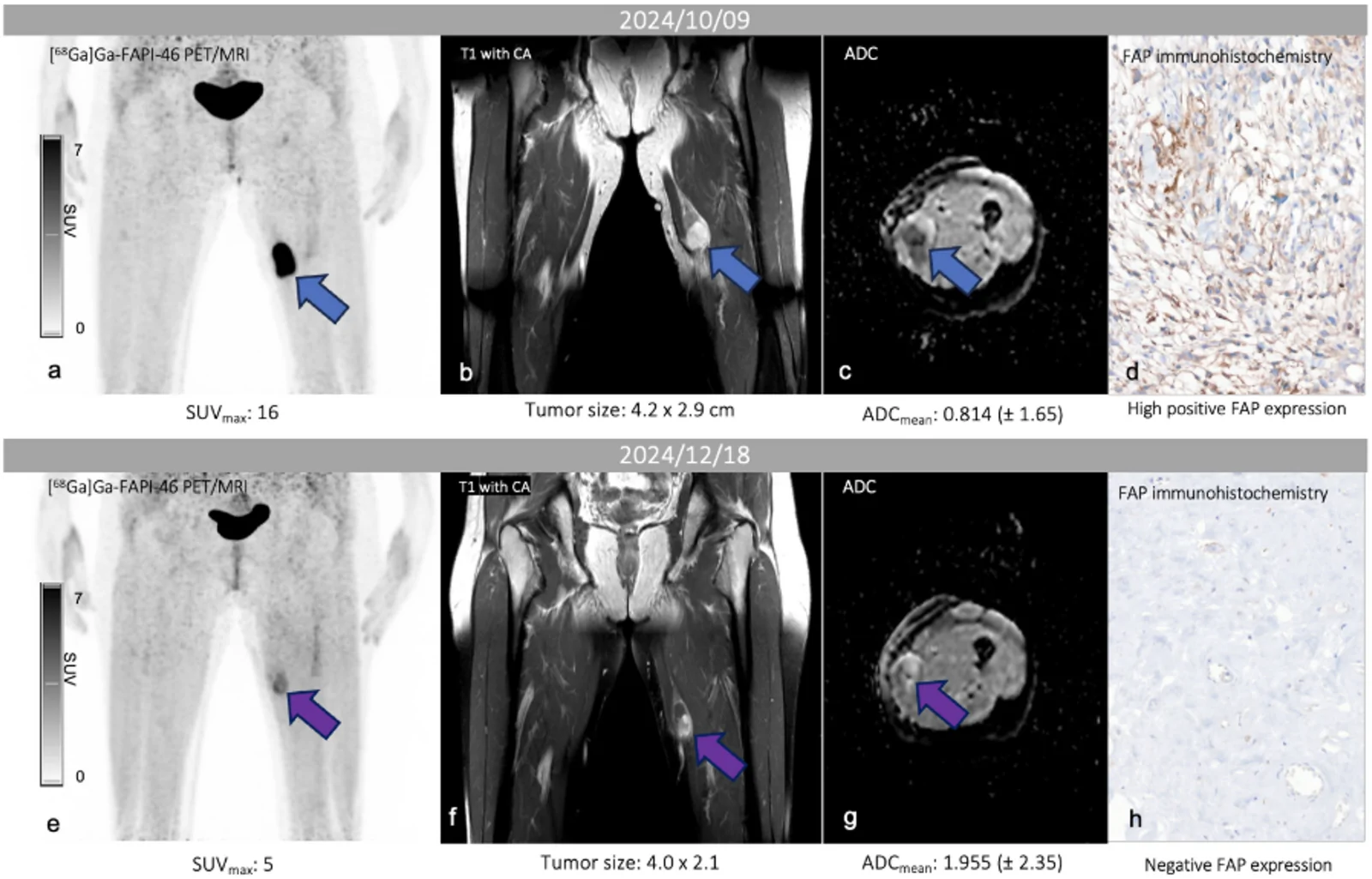
[^68^Ga]Ga‑FAPI‑46 PET/MRI before (**a-d**) and after (**e-h**) neoadjuvant radiotherapy of MFS of the right thigh. Imaging demonstrates a marked decrease in SUV_max_, with only slight reduction in tumor size, and an increase in *ADC*_*mean*_. Histopathological analysis revealed a regression rate of > 90%, indicating an excellent treatment response. Immunohistochemistry revealed a shift from high to low positive FAP expression

In two patients, imaging was performed both before and 12 weeks after neoadjuvant radiotherapy. The first patient (Fig. 6) had a high-grade retroperitoneal DDLPS with 13.5 × 8.2 cm and intense [^68^Ga]Ga-FAPI-46 uptake (SUV_max_ of 7.5) in baseline imaging. Post-radiotherapy, the tumor size decreased only slightly (SD, approx. 1 cm), while SUV_max_ was reduced by 40% to SUV_max_ 4.6 showing PMR. Concurrently, ADC_mean_ increased from 1.065 ± 0.88 to 1.428 ± 1.65 × 10⁻³ mm²/s, consistent with decreased cellularity and ultimately treatment response. The second patient (Fig. 7) with a primary MFS of the thigh with 4.2 × 2.9 cm received preoperative radiotherapy (50 Gy). Pretreatment PET/MRI demonstrated intense tracer uptake (SUV_max_ 16). Following radiotherapy, tumor size decreased slightly to 4.0 × 2.1 cm (SD), while SUV_max_ dropped by almost 70% to SUV_max_ 5.0 (PMR). Correspondingly, ADC_mean_ increased markedly from 0.814 ± 1.65 to 1.955 ± 2.35 × 10⁻³ mm²/s. Histopathological evaluation of the resected specimen showed > 90% tumor regression. Notably, immunohistochemistry revealed a shift from positive to negative FAP expression, suggesting treatment-induced stromal remodeling.

**Fig. 8:**
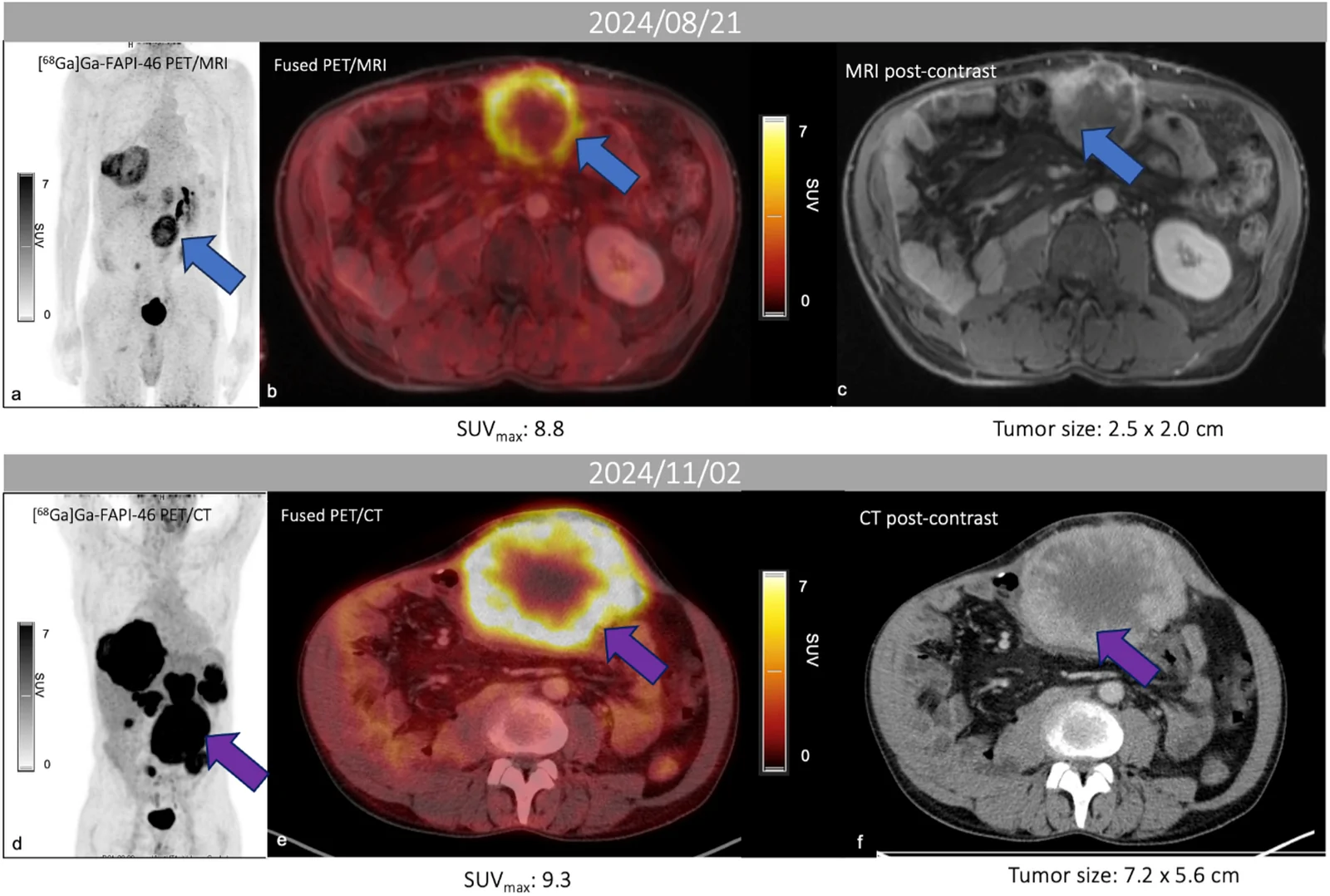
[^68^Ga]Ga‑FAPI‑46 PET/MRI before (**a-c**) and [^68^Ga]Ga‑FAPI‑46 PET/CT after (**d-f**) [^177^Lu]Lu-FAPI-46 treatment of a recurrent multifocal retroperitoneal DDLPS. The images demonstrate significant tumor progression under treatment, with significant increase in both lesion size and SUV_max_ with multiple newly delineated tumor lesions

One patient with recurrent multifocal retroperitoneal high-grade DDLPS exhibited significant [^68^Ga]Ga-FAPI-46 uptake (SUV_max_ 8.8) in a lesion infiltrating the abdominal wall (Fig. 8). Given the inoperability and marked tracer uptake on PET, the patient was treated with 2 cycles of [¹⁷⁷Lu]Lu-FAPI-46. Follow-up [^68^Ga]Ga-FAPI-46 PET/CT 4 weeks after end of therapy revealed significant disease progression with multiple new tumor lesions with high [^68^Ga]Ga-FAPI-46 uptake. The abdominal wall lesion increased in size from 2.5 × 2.0 cm to 7.2 × 5.6 cm (PD) with slightly increased SUV_max_ of 9.3 (PMD). Despite strong tracer accumulation on PET imaging, FAPI-targeted radioligand therapy did not achieve treatment response. The fourth patient had a recurrent dedifferentiated liposarcoma (G2) of the lower extremity and received two cycles of doxorubicin–vincristine. During follow-up, the recurrent lesion demonstrated progressive increase in size (PD) without a relevant change in [^68^Ga]Ga-FAPI-46 uptake (SMD; SUV_max_ 2.9).

## Discussion

This exploratory study evaluated integrated [^68^Ga]Ga-FAPI-46 PET/MRI for non-invasive tumor assessment in high-grade STS. Across all histological subtypes, visually detectable tracer uptake was observed, with high mean SUV_max_ values, whereas FAP expression on IHC was present in fewer than half of the tumors. Neither SUV_max_ nor ADC correlated with FNCLCC grading in this pilot study. Early metabolic changes in [^68^Ga]Ga-FAPI-46 uptake and ADC were observed in relation to treatment outcome. These findings, although preliminary, suggest that [^68^Ga]Ga-FAPI-46 PET/MRI may provide complementary information for response assessment.

[^68^Ga]Ga-FAPI-46 uptake was heterogeneous across sarcoma subtypes, with the widest SUV_max_ range (2.9–33) observed in dedifferentiated DDLPS, likely reflecting marked intratumoral heterogeneity. These findings are consistent with previous reports demonstrating broad variability of tracer uptake in STS [23, 21, 24]. Despite these results, no significant correlation between SUV_max_ and FNCLCC grade was observed. The lack of an observed association with FNCLCC grade should be interpreted cautiously. The small cohort size, limited variability in tumor grade, and potential discrepancies between imaging-derived parameters and histopathological sampling may have contributed to the negative findings and need to be validated in larger cohorts. Interestingly, SUV_max_ values were higher and more variable in G2 compared with G3 sarcomas, whereas G3 tumors demonstrated a narrower uptake range. This may reflect biological heterogeneity and limitations of biopsy-based grading. In most patients, definitive postoperative grading was unavailable due to neoadjuvant therapy or non-surgical management, necessitating reliance on core-needle biopsies, which are known to carry a substantial risk of undergrading [5, 6]. These observations support the clinical utility of dichotomous grading (low vs. high grade), particularly in the neoadjuvant setting, and are in line with previous reports showing limited ability of FAPI PET to distinguish sarcoma grades [25].

Correlation between tracer uptake and histopathological FAP expression has been reported previously, particularly in MFS, SFT, and UPS. In our cohort, FAP expression was detected in fewer than half of the tumors (2 DDLPS, 1 UPS, 1 MFS), with both positive and negative findings in DDLPS. This discordance likely reflects the pronounced intratumoral heterogeneity characteristic of STS, particularly in larger lesions. In addition, histopathological assessment is inherently limited by sampling bias, as only a small fraction of the tumor is analyzed and biopsy specimens may not correspond to the region with highest tracer uptake on imaging. Treatment-related stromal remodeling following prior therapy may further contribute to discrepancies between imaging and immunohistochemical findings. Moreover, differences between FAP expression measured ex vivo by immunohistochemistry and in vivo tracer uptake may be influenced by biological and pharmacokinetic factors beyond target expression alone. Taken together, PET-IHC discordance likely reflects the combined effects of tumor microenvironment heterogeneity and methodological differences between imaging and tissue-based assessment. Moreover, it should be noted that tissue samples were analyzed independently of the imaging findings; therefore, precise spatial matching between the imaging data and the corresponding tissue-sampling sites was not intended. At the same time, this approach reflects real-world clinical practice and provides preliminary, hypothesis-generating insights that may inform future investigations in larger and more homogeneous sarcoma cohorts.

In line with prior studies, tumors with strong FAP expression (IHC score 3) showed significantly higher SUVmax values, while PET-positive/IHC-negative cases were also observed [20]. Notably, immunohistochemistry revealed a shift from positive to negative FAP expression, suggesting treatment-induced stromal remodeling.

ADC values have previously been shown to differentiate benign from malignant soft tissue lesions and to correlate with tumor grade [26, 27]. Reported mean ADC values range from 1.08 ± 0.11 to 1.6 ± 0.59 × 10^-^³ mm²/s for low-grade sarcomas and from 0.79 ± 0.84 to 1.13 ± 0.23 × 10^-^³ mm²/s in high-grade sarcomas [8, 10, 28]. In our cohort, only G2 and G3 STS were analyzed, with mean ADC values of 0.921 ± 0.077 × 10^-^³ mm²/s for G2 and 0.945 ± 0.176 × 10^-^³ mm²/s for G3 STS, showing substantially overlap. These findings support the classification of G2 and G3 sarcomas as high-grade disease in clinical practice. Although ADC alone cannot reliably differentiate between these subgroups, it remains useful for binary grading and for distinguishing benign from malignant lesions [3, 29]. We further observed strong concordance between [^68^Ga]Ga-FAPI-46 uptake and DCE-MRI, suggesting that both modalities may reflect overlapping biological processes related to tumor vascularity and stromal activity [30].

A significant inverse correlation was observed between ADC_mean_ from functional MRI and SUV_max_ from FAPI PET. These preliminary observations indicate that tracer uptake, in addition to ADC, could potentially contribute to the functional characterization of STS. Confirmation of this potential role will require investigation in larger, adequately powered studies.

Intratumoral heterogeneity, a known feature of high-grade STS, was similarly depicted by PET and DCE-MRI, with areas of high tracer uptake corresponding to intense contrast enhancement [7, 21, 31].

To date, data on FAPI-targeted PET for therapy monitoring in STS are limited. In our study, four patients underwent [^68^Ga]Ga-FAPI-46 PET/MRI both before and after treatment. Notably, discrepancies in treatment response were found in three of four patients comparing mPERCIST with RECIST 1.1 criteria in these patients, with ADC and IHC results confirming response in PET, including concomitant changes in histopathology (e.g. necrosis) and ADC values compared to MRI alone. These observations could hint that PET may detect treatment response earlier than anatomical imaging; however, no definitive conclusions can be drawn given the small and heterogeneous subgroup in this study. Beyond its diagnostic role, FAP-targeted imaging has also emerged as a promising theranostic approach. Recent studies have reported encouraging initial clinical experience and good tolerability of [^177^Lu]Lu-FAPI-2286 therapy in a small cohort of patients with advanced metastatic malignancies, including sarcomas, supporting the potential of FAP-targeted radionuclide therapy [32, 33]. Conversely, one patient treated with [^177^Lu]Lu-FAPI-46 therapy in our cohort showed progressive disease despite high baseline uptake, underscoring that high tracer uptake alone may not reliably predict therapeutic benefit and improved biomarkers for patient selection in theranostic approaches [21, 31, 34].

The main limitation of this study is the small sample size within this pilot study, which should be taken into account when interpreting these findings. The small sample size limits the statistical power and precision of the estimates and increases the risk of both false-positive and unstable findings. Moreover the observed findings may have been influenced by the marked histopathological heterogeneity. As histopathological assessment was based on biopsy or resection specimens obtained from only part of the tumor, regional variations in FAP expression may not have been fully captured by the imaging-pathology correlation. Histopathological analyses were based on tumor samples considered representative by the treating pathologist, either from diagnostic core biopsies or surgical specimens. Owing to the study design, it could not be confirmed whether the analyzed tissue corresponded to the tumor region demonstrating the highest SUV_max_ on [^68^Ga]Ga-FAPI-46 PET/MRI. This limitation may have influenced the observed imaging–pathology correlations.

As an exploratory study, hypothesis-generating study, the generalizability of these findings to a broader sarcoma population remains limited. Further validation to define the role of integrated [^68^Ga]Ga-FAPI-46 PET/MRI in grading, response assessment, and therapy guidance in STS in larger, independent cohorts, ideally incorporating analyses of distinct sarcoma subtypes, is required to determine their broader applicability.

## Conclusion

In this exploratory pilot study, [^68^Ga]Ga-FAPI-46 PET/MRI provided complementary functional information in patients with high-grade soft-tissue sarcoma. While no association with FNCLCC grading was observed, an inverse correlation between ADC_mean_ and SUV_max_ was identified within this small cohort. In addition, preliminary observations suggested that early changes in tracer uptake and ADC may reflect treatment-related changes and could potentially be associated with treatment response. As these observations were derived from a small pilot cohort, the findings should be regarded as preliminary, and their generalizability to a broader sarcoma population remains limited. Larger prospective studies are needed to confirm these findings and to further define the potential role of [^68^Ga]Ga-FAPI-46 PET/MRI in treatment monitoring and response assessment.

## Data Availability

The datasets generated during and/or analyzed during the current study are available from the corresponding author on reasonable request.

## Abbreviations

ADC: Apparent diffusion coefficient
CAFs: Cancer-associated fibroblasts
CT: Computed tomography
CI: Confidence interval
DDLPS: Dedifferentiated liposarcoma
DWI: Diffusion-weighted imaging
DCE-MRI: Dynamic contrast enhanced magnetic resonance imaging
FAP: Fibroblast Activation Protein
FAPI: Fibroblast Activation Protein Inhibitor
[^18^F]FDG: ^18^Fluorodeoxyglucose
FNCLCC: Fédération Nationale des Centres de Lutte Contre le Cancer
G: Grade
[^68^Ga]Ga‑FAPI‑46: ^68^Gallium Fibroblast Activation Protein Inhibitor 46
IHC: Immunohistochemistry
LR: Local recurrence
LMS: Leiomyosarcoma
LPS: Liposarcoma
MRI: Magnetic resonance imaging
MFS: Myxofibrosarcoma
PET: Positron emission tomography
PT: Primary tumor
ROI: Region of interest
SD: Standard deviation
STS: Soft tissue sarcoma
SFT: Solitary fibrous tumor
SUV: Standardized uptake value
UPS: Undifferentiated pleomorphic sarcoma

## References

[1] Ressing M, et al. Strengthening health data on a rare and heterogeneous disease: sarcoma incidence and histological subtypes in Germany. BMC Public Health. 2018;18(1):235. 29433465 10.1186/s12889-018-5131-4 PMC5809940

[2] Callegaro D, et al. Development and external validation of two nomograms to predict overall survival and occurrence of distant metastases in adults after surgical resection of localised soft-tissue sarcomas of the extremities: a retrospective analysis. Lancet Oncol. 2016;17(5):671–80. 27068860 10.1016/S1470-2045(16)00010-3

[3] Casali PG, et al. Soft tissue and visceral sarcomas: ESMO–EURACAN Clinical Practice Guidelines for diagnosis, treatment and follow-up. Ann Oncol. 2018;29(Supplement4):iv51–67. 29846498 10.1093/annonc/mdy096

[4] Gielen JLMA, et al. Accuracy of MRI in characterization of soft tissue tumors and tumor-like lesions. A prospective study in 548 patients. Eur Radiol. 2004;14(12):2320–30. 15290067 10.1007/s00330-004-2431-0

[5] Schneider N, et al. The Adequacy of Core Biopsy in the Assessment of Smooth Muscle Neoplasms of Soft Tissues: Implications for Treatment and Prognosis. Am J Surg Pathol. 2017;41(7):923–31. 28505003 10.1097/PAS.0000000000000867

[6] Almond LM, et al. Diagnostic accuracy of percutaneous biopsy in retroperitoneal sarcoma. Br J Surg. 2019;106(4):395–403. 30675910 10.1002/bjs.11064

[7] Crombé A, et al. Soft-Tissue Sarcomas: Assessment of MRI Features Correlating with Histologic Grade and Patient Outcome. Radiology. 2019;291(3):710–21. 30964422 10.1148/radiol.2019181659

[8] Chhabra A, et al. Conventional MR and diffusion-weighted imaging of musculoskeletal soft tissue malignancy: correlation with histologic grading. Eur Radiol. 2019;29(8):4485–94. 30511176 10.1007/s00330-018-5845-9

[9] Schmitz F, Sedaghat S. Inferring malignancy grade of soft tissue sarcomas from magnetic resonance imaging features: A systematic review. Eur J Radiol. 2024;177:111548. 38852328 10.1016/j.ejrad.2024.111548

[10] Hettler M, et al. Comparing Apparent Diffusion Coefficient and FNCLCC Grading to Improve Pretreatment Grading of Soft Tissue Sarcoma-A Translational Feasibility Study on Fusion Imaging. Cancers (Basel). 2022;14(17):4431. 36077866 10.3390/cancers14174331 PMC9454612

[11] Annovazzi A, et al. Diagnostic and Clinical Impact of 18F-FDG PET/CT in Staging and Restaging Soft-Tissue Sarcomas of the Extremities and Trunk: Mono-Institutional Retrospective Study of a Sarcoma Referral Center. J Clin Med. 2020;9(8):2549. 32781683 10.3390/jcm9082549 PMC7463806

[12] Parghane RV, Basu S. Dual–time point 18 F-FDG-PET and PET/CT for Differentiating Benign From Malignant Musculoskeletal Lesions: Opportunities and Limitations. Semin Nucl Med. 2017;47(4):373–91. 28583277 10.1053/j.semnuclmed.2017.02.009

[13] Gu B, et al. Head-to-head evaluation of [18F]FDG and [68 Ga]Ga-DOTA-FAPI-04 PET/CT in recurrent soft tissue sarcoma. Eur J Nucl Med Mol Imaging. 2022;49(8):2889–901. 35113192 10.1007/s00259-022-05700-4 PMC9206606

[14] Dohi O, et al. Histogenesis-specific expression of fibroblast activation protein and dipeptidylpeptidase‐IV in human bone and soft tissue tumours. Histopathology. 2009;55(4):432–40. 19817894 10.1111/j.1365-2559.2009.03399.x PMC2784039

[15] Rettig WJ, et al. Cell-surface glycoproteins of human sarcomas: differential expression in normal and malignant tissues and cultured cells. Proc Natl Acad Sci. 1988;85(9):3110–4. 2896356 10.1073/pnas.85.9.3110 PMC280153

[16] Pabst KM, et al. [68Ga]Ga-FAPI-46 PET accuracy for cancer imaging with histopathology validation: a single-centre, single-arm, interventional, phase 2 trial. Lancet Oncol. 2025;26(9):1204–14. 40774265 10.1016/S1470-2045(25)00299-2

[17] Giesel FL, et al. 68Ga-FAPI PET/CT: Biodistribution and Preliminary Dosimetry Estimate of 2 DOTA-Containing FAP-Targeting Agents in Patients with Various Cancers. J Nucl Med. 2019;60(3):386–92. 30072500 10.2967/jnumed.118.215913 PMC6424229

[18] Kratochwil C, et al. 68-Ga-FAPI PET/CT: Tracer Uptake in 28 Different Kinds of Cancer. J Nucl Med. 2019;60(6):801–5. 30954939 10.2967/jnumed.119.227967 PMC6581228

[19] Scanlan MJ, et al. Molecular cloning of fibroblast activation protein alpha, a member of the serine protease family selectively expressed in stromal fibroblasts of epithelial cancers. Proc Natl Acad Sci. 1994;91(12):5657–61. 7911242 10.1073/pnas.91.12.5657 PMC44055

[20] Kessler L, et al. 68Ga-FAPI as a Diagnostic Tool in Sarcoma: Data from the 68Ga-FAPI PET Prospective Observational Trial. J Nucl Med. 2022;63(1):89–95. 33931468 10.2967/jnumed.121.262096 PMC8717183

[21] Lanzafame H, et al. 68Ga-Fibroblast Activation Protein Inhibitor PET/CT Improves Detection of Intermediate and Low-Grade Sarcomas and Identifies Candidates for Radiopharmaceutical Therapy. J Nucl Med. 2024;65(6):880–7. 38724279 10.2967/jnumed.123.267248

[22] Henry LR, et al. Clinical Implications of Fibroblast Activation Protein in Patients with Colon Cancer. Clin Cancer Res. 2007;13(6):1736–41. 17363526 10.1158/1078-0432.CCR-06-1746

[23] Abdulrahman M, et al. Exploring the Diagnostic and Theranostic Role of FAPI PET in Soft Tissue Sarcoma: A Systematic Review. Nuclear Medicine and Molecular Imaging; 2025. 10.1007/s13139-025-00936-y PMC12446160 40979140

[24] Chen H, et al. Comparison of [68Ga]Ga-DOTA-FAPI-04 and [18F] FDG PET/CT for the diagnosis of primary and metastatic lesions in patients with various types of cancer. Eur J Nucl Med Mol Imaging. 2020;47(8):1820–32. 32222810 10.1007/s00259-020-04769-z

[25] Koerber SA, et al. Novel FAP ligands enable improved imaging contrast in sarcoma patients due to FAPI-PET/CT. Eur J Nucl Med Mol Imaging. 2021;48(12):3918–24. 34018010 10.1007/s00259-021-05374-4 PMC8484190

[26] Pekcevik Y, Onur M, Kahya, Kaya A. Characterization of Soft Tissue Tumors by Diffusion-Weighted Imaging. Iran J Radiol. 2015;12(3):e15478. 26557269 10.5812/iranjradiol.15478v2 PMC4632135

[27] Gondim Teixeira PA, et al. Diffusion-weighted magnetic resonance imaging for the initial characterization of non-fatty soft tissue tumors: correlation between T2 signal intensity and ADC values. Skeletal Radiol. 2016;45(2):263–71. 26619837 10.1007/s00256-015-2302-6

[28] Razek A, et al. Assessment of soft tissue tumours of the extremities with diffusion echoplanar MR imaging. Radiol Med. 2012;117(1):96–101. 21744251 10.1007/s11547-011-0709-2

[29] Machado I, et al. Ki-67 immunoexpression and radiological assessment of necrosis improves accuracy of conventional and modified core biopsy systems in predicting the final grade assigned to adult-soft tissue sarcomas. An international collaborative study. Pathol Res Pract. 2021;225:153562. 34329836 10.1016/j.prp.2021.153562

[30] Zhang Y, et al. Diagnostic Performance of Dynamic Contrast-Enhanced MRI and 18F-FDG PET/CT for Evaluation of Soft Tissue Tumors and Correlation with Pathology Parameters. Acad Radiol. 2022;29(12):1842–51. 35396157 10.1016/j.acra.2022.03.009

[31] Lanzafame H, et al. 90Y-FAPI-46 Theranostics Leads to Near-Complete Metabolic Response in 3 Patients with Solitary Fibrous Tumors. J Nucl Med. 2025;66(9):1378–84. 40707141 10.2967/jnumed.125.269572

[32] Banihashemian SS, et al. Feasibility and therapeutic potential of [177Lu]Lu-FAPI-2286 in patients with advanced metastatic sarcoma. Eur J Nucl Med Mol Imaging. 2024;52(1):237–46. 39060377 10.1007/s00259-024-06795-7

[33] Banihashemian SS, et al. First Experience of Radionuclide Therapy With 177Lu-FAPI-2286 in a Patient With Metastatic Mediastinal Sarcoma. Clin Nucl Med. 2024;49(7):e334–7. 38831513 10.1097/RLU.0000000000005255

[34] Assadi M, et al. Feasibility and Therapeutic Potential of 177Lu–Fibroblast Activation Protein Inhibitor–46 for Patients With Relapsed or Refractory Cancers. Clin Nucl Med. 2021;46(11):e523–30. 34269729 10.1097/RLU.0000000000003810

